# Assessing the inequality in out-of-pocket health expenditure among the chronically and non-chronically ill in Saudi Arabia: a Blinder-Oaxaca decomposition analysis

**DOI:** 10.1186/s12939-022-01810-5

**Published:** 2022-12-31

**Authors:** Mohammed Khaled Al-Hanawi, Purity Njagi

**Affiliations:** 1 Department of Health Services and Hospital Administration, Faculty of Economics and Administration, King Abdulaziz University, Jeddah, Saudi Arabia; 2 Health Economics Research Group, King Abdulaziz University, Jeddah, Saudi Arabia; 3 African Renaissance, Nairobi, Kenya

**Keywords:** Out-of-Pocket, Inequality, Chronic illness, Saudi Arabia, Blinder-Oaxaca decomposition

## Abstract

**Background:**

Many high-income countries have made significant progress towards achieving universal health coverage. Nevertheless, out-of-pocket (OOP) health expenditure continues to undermine the effectiveness of the universal healthcare system. In Saudi Arabia, due to the overburdened free public health services, many people opt for alternative healthcare services, risking high OOP payments. The presence of chronic illness further exacerbates this situation. However, there is limited evidence on the extent of the gap in OOP health expenditure between the chronically and non-chronically ill and the associated drivers contributing to this gap. The aim of this study was to assess inequalities in relative OOP health expenditure, estimated as the percentage of income spent on healthcare, between the chronically and non-chronically ill in Saudi Arabia and their associated drivers.

**Methods:**

Data from 10,785 respondents were obtained from a national cross-sectional survey conducted in Saudi Arabia as part of the 2018 Family Health Survey. Inequalities in relative OOP health expenditure were measured using concentration indices and curves. A Blinder-Oaxaca decomposition analysis was used to assess the differences in relative OOP health expenditure between the chronically and non-chronically ill.

**Results:**

The results showed that the chronically ill experience a higher financial burden due to healthcare services in absolute costs and relative to their income compared to the non-chronically ill. In addition, there was higher pro-poor inequality (–0.1985) in relative OOP health expenditure among the chronically ill compared to that (–0.1195) among the non-chronically ill. There was a 2.6% gap in relative OOP health expenditure among the chronically and non-chronically ill, of which 53.8% was attributable to unexplained factors, with explained factors accounting for the 46.2% difference. Factors that significantly contributed to the overall gap (i.e. both explained and non-explained factors) included employment status, insurance status, self-rated health, and periodic check-ups.

**Conclusion:**

This study underscores the high financial burden due to OOP payments among the chronically ill and the existence of pro-poor inequalities. In addition, there is a significant gap in relative OOP health expenditure between the chronically and non-chronically ill, which is mainly attributable to differences in socio-economic characteristics. This indicates that the existing financial mechanisms have not been sufficient in cushioning the chronically ill and less well off in Saudi Arabia. This situation calls for health policymakers to integrate a social safety net into the health financing system and to prioritize the disadvantaged population, thereby ensuring access to health services without experiencing financial hardship.

## Introduction

The United Nations Sustainable Development Goals (SDGs) promote the achievement of universal health coverage (UHC), which means that all people have access to the quality health services they need with minimal exposure to financial hardship [1, 2]. Over recent decades, several governments have sought to improve the health of the population while reducing the associated financial burden. However, the presence of universal healthcare access and extensive coverage does not completely eliminate the requirement for out-of-pocket (OOP) payments to receive healthcare goods and services [3]. OOP health expenditure refers to all payments spent on medical costs such as consultation fees, medicine, diagnosis, laboratory, radiology, and admission, along with other non-medical expenses such as transportation [4, 5]. Thus, ongoing monitoring of OOP healthcare expenditure is an essential part of assessing health system performance, even in countries with UHC.

OOP health expenditure is the primary source of healthcare financing in many low- and middle-income countries (LMICs) [6]. However, OOP health expenditure is also recorded in high-income countries given that some healthcare services are still regularly paid through OOP due to the overburdening of the free public healthcare system [5]. The increase in OOP health expenditure in high-income countries undermines the effectiveness of the purported universal healthcare system. It is argued that OOP health expenditure is largely regressive and ineffective due to health differentials based on socio-economic characteristics [6]. Among the poor, OOP health expenditure likely leads to catastrophic health expenditure, where health expenditure exceeds a certain threshold of household consumption [7]. Knowledge on the inequalities in OOP health expenditure can help to shape pro-poor interventions. Without accurate knowledge, the design of such interventions will be imperfectly informed and ineffective.

Differentials in OOP health expenditure exist between the chronically ill and the population without chronic illness. Due to the permanent and increased healthcare consumption, the burden of OOP health expenditure is reportedly high among individuals with chronic illness. Those who develop a chronic disease have a higher chance of leaving the workforce, thereby experiencing a decline in wages associated with paid employment [2]. Moreover, chronic illness may lead to disability, early retirement, or other types of dependence on social security systems. Additionally, the chronically ill have less generous health insurance coverage than the non-chronically ill as treatments for chronic conditions are usually of high cost [8]. These factors constrain household budgets, leading to a significant increase in the financial burden for households and individuals with chronic illness [3].

Studies have shown that having household members with chronic illnesses is one of the factors that increases the likelihood of incurring OOP health expenditures [6, 9–14]. Despite multiple lines of evidence showing an increased burden of OOP health expenditure on the chronically ill, no studies have endeavoured to assess the factors that drive the gap and inequality in the OOP health expenditure between the chronically and non-chronically ill. Assessing such a gap is important given the rise in the proportion of the population with chronic illness as the population ages. Al-Hanawi [15] stresses the existence of a shift in epidemiological progression of diseases from predominantly communicable diseases towards chronic non-communicable diseases, which now represent the leading cause of premature death worldwide [16].

Based on this context, the aim of this study was to contribute to and expand the existing literature by assessing inequalities in relative OOP health expenditure, estimated as the percentage of income spent on healthcare, between the chronically and non-chronically ill and the associated drivers in the Kingdom of Saudi Arabia (KSA). These findings will establish the gap in OOP health expenditure between these two groups and the factors that contribute to this gap. This study is intended to inform financing mechanisms in considering individuals with chronic illness and the associated factors leading to their OOP health expenditure, given that some sub-groups are more vulnerable than others.

The KSA is a relevant context to conduct this study for several reasons. Firstly, free public healthcare services in the KSA are overburdened and overcrowded, thereby forcing many Saudis to use private healthcare services; currently, OOP expenditures are estimated to account for approximately 14% of the total health expenditure [17–19]. Secondly, despite the country providing free healthcare services to its citizens through public health facilities, approximately 56% of the workforce in the KSA relies on private health insurance and OOP expenditure [20]. Thirdly, there is a greater prevalence of chronic diseases, especially non-communicable diseases, in the KSA, compared to other countries in the Arabian Gulf region [15]. Since the healthcare system occupies the largest share of the national budget, the multiplicity of chronic illness exerts continued pressure on the government resource envelope; thus, this study is also of welfare significance as it has policy implications that cut across health, social, and economic aspects relevant to the country.

## Materials and methods

### Data source

This study used self-weighted data from the national representative Family Health Survey (FHS) conducted by the General Authority for Statistics (GaStat) in 2018 in collaboration with several actors in the KSA, including the Ministry of Health (MOH), Saudi Health Council, as well as the private and academic sectors [21]. This nationally representative survey covers the 13 administrative regions of the country and collects information relating to geography, basic characteristics of household members, family income and expenditure, marriage and family planning, fertility and mortality, and health status of individuals, including whether or not they suffer from any chronic diseases, among other topics. The FHS collected a total sample of 15,265 responses randomly selected across all 13 regions of the KSA. For this study, the analysis was limited to respondents who were aged 18 years or older, and provided complete information on all variables of interest, resulting in a sample of 10,785 respondents.

### Variables

The survey asked respondents to report their average household monthly OOP expenditure on health and their average household monthly income (in Saudi Riyal [SR]). The outcome variable of interest in this study is the relative OOP health expenditure, estimated as the percentage of income spent on healthcare. OOP health expenditure relates to direct medical costs paid by individuals to access healthcare services, including medicines, consultations, diagnosis, tests, laboratory, radiology, and admission, along with non-medical costs such as transport for both inpatients and outpatients, among others. The Saudi FHS survey expresses OOP expenditure as the total costs paid for healthcare services and does not discriminate whether any or all of the amount has been reimbursed by private health insurance; hence, caution must be exercised in interpreting the outcome, which represents the general OOP health expenditure rather than the net OOP health expenditure that could also include unobservable reimbursements.

The exploratory variables (covariates) are informed by Andersen’s Health Behaviour model, distinguishing the following three categories of factors affecting access to healthcare services [22]: (i) *enabling factors*, including gender, age group, and marital status; (ii) *predisposing factors*, including educational level, economic status, employment status, and health insurance coverage status; and (ii) *need factors*, including periodic check-ups and self-rated health. In addition, these socioeconomic factors are based on evidence from previous studies that demonstrated their relative influence on OOP health expenditure [23–25].

### Statistical analysis

#### Difference in OOP expenditure by background characteristics

We calculated the absolute mean OOP health expenditure for each group (with and without chronic illness) separately. We then calculated the relative OOP health expenditure, estimated as the percentage of income spent on healthcare, across the two groups. We further analysed the difference in absolute and relative OOP health expenditure across the socioeconomic covariates.

#### Inequality in relative OOP health expenditure

We used the concentration curve and index to gauge the extent of inequality in mean relative OOP between individuals with and without chronic illness. The generalized concentration index was used to gauge the extent of inequality in relative OOP health expenditure between the two groups [26]. The concentration index has been frequently used to measure relative inequality in one health variable over the distribution of a socioeconomic variable [27]. To create a variable that ranks households from the poorest to the richest, we used the income to classify households into wealth quintiles.

The concentration index was then derived as follows:

1 $$\begin{array}{c}\mathrm{CI}= \frac{2}{{\upmu }_{\mathrm{h}}}\sum_{\mathrm{i}=1}^{\mathrm{n}}\left({\mathrm{h}}_{\mathrm{i}}-{\upmu }_{\mathrm{h}}\right)\left({\mathcal{R}}_{\mathrm{i}}- \frac{1}{2}\right)\\ =\frac{2}{{\upmu }_{\mathrm{h}}}\mathrm{cov}\left(\mathrm{h}, \mathcal{R}\right)\end{array}$$

where n denotes the number of observations, h_i_ is the health variable, μ is the mean of h, and ${\mathrm{R}}_{i}-\frac{1}{2}$ is the fractional socioeconomic rank, ranging from the poorest to the richest [28].

#### Blinder-Oaxaca decomposition

Given the difference in relative OOP health expenditure across individuals with and without chronic illness, we further ascertained the factors that drive this difference. Toward this end, we applied the Blinder-Oaxaca (B-O) decomposition approach, which has been extensively used to assess differences in health outcomes between two groups [28]. First, we measured the difference in the mean relative OOP between the chronically and non-chronically ill. Second, we applied B-O decomposition models [29] to examine how much of the observed gap can be explained by the differences in the characteristics of the groups by decomposing the differences into two components: one that is explained by the effect of the covariates (covariate effects/explained component) and another that is explained by the difference in the effect of the covariates (coefficient effects/unexplained component) [30].

This decomposition is represented as follows:

2 $$\mathcal{R}=\mathrm{\rm E}\left({X}_{A}\right) -\mathrm{\rm E}\left({X}_{B}\right)$$

where E(X) denotes the expected relative OOP accounted for by the group differences, A refers to one group (chronically ill), and B refers to the other group (non-chronically ill). The two-fold decomposition is divided into two components as follows:

3 $$R=Explained(Q) + Unexplained (U)$$

4 $$R={\lfloor E\left({X}_{A}\right)-E\left({X}_{B}\right)\rfloor}^{\mathrm{^{\prime}}} {\beta }^{*}+ \left[E{\left({X}_{A}\right)}^{\mathrm{^{\prime}}}\left({\beta }_{A}-\beta \right)+E{\left({X}_{B}\right)}^{\mathrm{^{\prime}}}\left({\beta }^{*}-{\beta }_{B}\right)\right]$$

where the first component is attributed to the group differences in the predictors (endowment/explained effect), as follows:

5 $$Q=\lfloor\mathrm{\rm E}\left({X}_{A}\right)-\mathrm{\rm E}\left({X}_{B}\right)\rfloor\mathrm{^{\prime}} {\beta }^{*}$$

The second component of Eq. (4) is the contribution of the difference in the coefficients (coefficient/unexplained effect), as follows:

6 $$U=\mathrm{\rm E}\left({X}_{A}\right)\mathrm{^{\prime}} ({\beta }_{A}-\beta )+E({X}_{B})\mathrm{^{\prime}} ({\beta }^{*}-{\beta }_{B})$$

### Ethical clearance

This study was based on the use of secondary data from the FHS, which was conducted, commissioned, funded, and managed in 2018 by GaStat that was in charge of all ethical procedures. All procedures performed in this study involving human participants complied with the institutional and/or national research committee ethical standards, and with the 1964 Helsinki Declaration and subsequent amendments or equivalent ethical standards. Informed consent was obtained from all participants. All personal identifiers were removed from the dataset by GaStat to allow for secondary data use. GaStat granted permission to use the data and thus no further clearance was necessary as this was performed at the data collection phase.

## Results

### Descriptive analysis

Table 1 shows the distribution of the study characteristics among the chronically and non-chronically ill groups. Overall, of the 10,785 sample respondents, the majority (*n* = 7,121, 66.03%) were not chronically ill, whereas 3,664 (33.97%) were chronically ill. The descriptive characteristics of the respondents are classified according to predisposing, enabling, and need factors that influence access to healthcare services. The predisposing factors include gender, age group, and marital status. Most of the respondents were male; this distribution was similar between the chronically and non-chronically ill, with more than half of the respondents being male. The majority of chronically ill were concentrated among the older age groups such that the proportion of chronically ill respondents increased with age, and the opposite trend was found for the non-chronically ill group. More than half of the respondents, overall and for each group, were married.

**Table 1:** Description of the study population characteristics

Characteristics	Categories	Chronically ill		Non-chronically ill		Total	
		**N**	**%**	**N**	**%**	**N**	**%**
**Predisposing factors**							
Gender	Male	1910	52.10	3946	55.43	5856	54.30
	Female	1754	47.90	3175	44.57	4929	45.70
Age group	18–29 years	146	3.98	2844	39.94	2990	27.72
	30–39 years	216	5.90	2182	30.64	2398	22.23
	40–49 years	574	15.67	1231	17.29	1805	16.74
	50–59 years	990	27.02	579	8.13	1569	14.55
	≥ 60 years	1738	47.43	285	4.00	2023	18.76
Marital status	Unmarried	803	21.92	2591	36.39	3394	31.47
	Married	2861	78.08	4530	63.61	7391	68.53
**Enabling factors**							
Education level	Below primary school	1427	38.95	786	11.04	2213	20.52
	Primary school	542	14.79	586	8.23	1128	10.46
	Intermediate school	539	14.71	786	11.04	1325	12.29
	Secondary school	487	13.25	2631	36.95	3118	28.91
	Pre-university diploma	209	5.70	500	7.02	709	6.57
	Higher education	460	12.55	1832	25.73	2292	21.25
Economic status	Poorest quintile	757	20.66	1537	21.58	2294	21.27
	Second quintile	765	20.88	1292	18.14	2057	19.07
	Middle quintile	699	19.08	1444	20.28	2143	19.87
	Fourth quintile	780	21.29	1722	24.18	2502	23.20
	Richest quintile	663	18.09	1126	15.81	1789	16.59
Employment status	Unemployed	1739	47.50	3337	46.90	5076	47.10
	Employed	852	23.20	3516	49.30	4368	40.50
	Retired	1073	29.30	268	3.80	1341	12.40
Insurance status	Not insured	2609	71.21	4644	65.22	7253	67.25
	Insured	1055	28.79	2477	34.78	3532	32.75
**Need factors**							
Self-rated health	Very bad or bad	476	12.99	18	0.25	494	4.58
	Mediocre	1085	29.61	82	1.15	1167	10.82
	Good or very good	2103	57.40	7021	98.60	9124	84.60
Periodic check-ups	No periodic check-ups	454	12.39	4780	67.13	5234	48.53
	Periodic check-ups	3210	87.61	2341	32.87	5551	51.47
**Total**		3664	33.97	7121	66.03	10,785	

Under enabling factors, respondents were categorized by education level, and economic, employment, and insurance status. Regarding education level, the majority (28.91%) of the respondents had a secondary level education. The majority of the chronically ill were unemployed (47.50%), whereas the majority of non-chronically ill were employed (49.30%). In addition, while the majority of the respondents were uninsured (67.25%), the proportion of uninsured chronically ill (71.21%) was higher compared to that of the uninsured non chronically ill (65.22%).

The need factors explored in this study included self-rated health and periodic check-ups. On average, most respondents had good or very good self-rated health; however, among the chronically ill, 12.99% rated their health as bad or very bad compared to only 0.25% among the non-chronically ill. The great majority of the non-chronically ill (98.60%) rated their health as good or very good. Periodic check-ups were higher among chronically ill (87.61%), while the majority (67.13%) of the non-chronically ill respondents reported having no periodic check-ups.

### Average absolute and relative OOP expenditure across socioeconomic characteristics

Table 2 presents the distribution of average absolute and relative OOP health expenditure among the chronically and non-chronically ill across socioeconomic characteristics. We found differences in both absolute and relative OOP health expenditure between the chronically and non-chronically ill. Overall, the chronically ill had a higher mean OOP health expenditure of 658.75 SR than that of the non-chronically ill of 444.98 SR. In addition, the chronically ill spent 7.6% of their income on health compared to 5.0% spent by the non-chronically ill. Women had a higher absolute and relative OOP health expenditure than men for both the chronically and non-chronically ill groups. Among the chronically ill, those aged 18–29 years had higher absolute and relative OOP health expenditure at 865.62 SR and 8.7%, respectively. However, among the non-chronically ill, those aged 18–29 years still had the highest absolute OOP health expenditure at 493.37 SR, while those aged 60 years and older had the highest relative OOP health expenditure at 6.1%. The married group had the lowest relative OOP health expenditure at 7.4% for the chronically ill and 4.5% for the non-chronically ill. Notably, for each educational level category, the chronically ill had a higher absolute and relative OOP health expenditure than those of the non-chronically ill.

**Table 2:** Average absolute and relative OOP health expenditure

Characteristic	Categories	Chronically ill		Non-chronically ill	
		**Absolute** **OOP**	**Relative** **OOP**	**Absolute** **OOP**	**Relative** **OOP**
**Predisposing factors**					
Gender	Male	649.06	7.2%	378.25	4.5%
	Female	669.30	8.2%	527.92	5.7%
Age group	18–29 years	865.62	8.7%	493.37	5.6%
	30–39 years	626.93	7.5%	376.22	4.3%
	40–49 years	557.89	8.3%	443.92	4.7%
	50–59 years	627.79	7.1%	491.51	5.3%
	≥ 60 years	696.27	7.7%	398.61	6.1%
Marital status	Unmarried	551.30	8.6%	538.04	6.0%
	Married	688.91	7.4%	391.75	4.5%
**Enabling factors**					
Education level	Below primary school	567.56	8.6%	295.04	5.5%
	Primary school	642.97	9.0%	315.36	5.3%
	Intermediate school	670.59	7.2%	372.75	5.5%
	Secondary school	677.27	6.2%	456.34	5.1%
	Pre university diploma	970.73	6.2%	472.42	3.8%
	Higher education	785.00	5.7%	557.96	4.8%
Economic status	Poorest quintile	321.70	11.4%	164.71	6.3%
	Second quintile	490.07	9.4%	315.01	5.9%
	Middle quintile	506.82	6.4%	401.66	5.0%
	Fourth quintile	799.28	6.4%	543.96	4.4%
	Richest quintile	1233.07	4.1%	880.86	3.4%
Employment status	Unemployed	706.60	8.6%	501.35	5.8%
	Employed	567.79	6.9%	389.00	4.2%
	Retired	642.09	6.4%	550.81	5.8%
Insurance status	Not insured	656.56	8.9%	520.05	5.8%
	Insured	664.16	4.6%	304.24	3.6%
**Need factors**					
Self-rated health	Very bad or bad	842.14	8.9%	561.11	19.6%
	Mediocre	637.52	8.5%	485.12	9.8%
	Good or very good	628.19	6.9%	444.21	4.9%
Period check-ups	No periodic check-ups	657.85	9.2%	376.41	4.7%
	Periodic check-ups	658.88	7.4%	585.00	5.7%
**Average**		658.75	7.6%	444.98	5.0%

As shown in Table 2, on average, among the chronically ill, the richest had a significantly higher OOP health expenditure at 1233.07 SR compared to only 321.70 SR paid by the poorest. However, as a proportion of income, OOP health expenditure accounted for 11.4% of the income of the poor, whereas this proportion was only 4.1% for the richest group. This was similar to the discrepancy observed among the non-chronically ill, whereby the mean OOP health expenditure increased, and the relative OOP decreased when moving up to the wealth quintile. In addition, the unemployed and retired have greater absolute OOP health expenditure compared to that of the employed for both the chronically and non-chronically ill. Individuals who rated their health as very bad or bad had the highest absolute and relative OOP health expenditure for both the chronically and non-chronically ill. Among the chronically ill, there was no difference in mean OOP health expenditure between those who had periodic check-ups and those who did not. However, those that did not go for periodic check-ups had a higher relative OOP health expenditure of 9.2% compared to 7.4% among those with periodic check-ups.

### Inequality in relative OOP health expenditure between the chronically and non-chronically ill

Analysis of the inequality in relative OOP health expenditure between the chronically and non-chronically ill in the KSA showed a pro-poor distribution, meaning that the inequalities are concentrated amongst the less well-off. In other words, the poor spend a higher share of their income on health than the well-off. This inequality was higher among the chronically ill (–0.198) than the non-chronically ill (–0.119). This implies that the poor with a chronic illness experience even higher relative OOP health expenditure compared to that of the poor who are not chronically ill. In addition, there was a –0.078 significant difference in the inequality between the chronically and non-chronically ill, as shown in Table 3.

**Table 3:** Wagstaff concentration index between the chronically and non-chronically ill

Illness	No. of observations	Index value	Robust Std. error	*p*-value
No chronic illness (A)	7121	–0.1195	0.0113	< 0.001
Chronic illness (B)	3664	–0.1985	0.0143	< 0.001
CI_B_ – CI_A_		–0.078	0.018	< 0.001

**Fig. 1:**
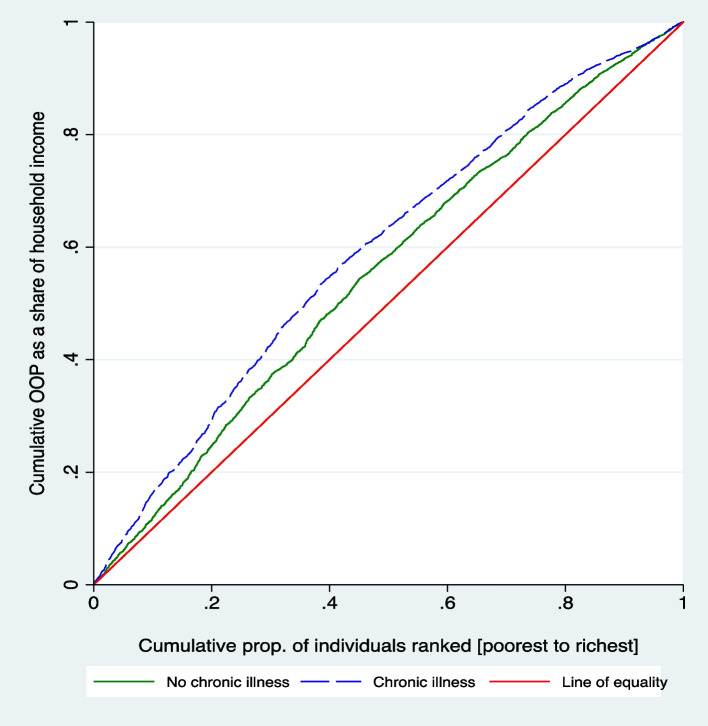
Concentration curves for the chronically and non-chronically ill

Figure 1 presents the concentration curves for the chronically and non-chronically ill. Both curves lay above the line of equality (red), indicating pro-poor inequality in relative OOP health expenditure for both groups. In addition, the chronically ill curve is further from the line of equality compared to the non-chronically ill curve, supporting that inequality among the chronically ill is higher than the non-chronically ill.

### Blinder-Oaxaca decomposition of the gap in OOP health expenditure

We applied a Blinder-Oaxaca decomposition to explain the factors contributing to the observed difference in the mean gap of relative OOP health expenditure between the chronically and non-chronically ill. As summarized in Table 4, the analyses indicated that the relative OOP health expenditure is higher among individuals with a chronic illness (7.6%) compared with that of individuals without a chronic illness (5.0%), and the difference of 2.6% was statistically significant (*p* < 0.001). The bulk of the gap of 53.8% (–0.014/–0.026) in relative OOP health expenditure between the chronically and non-chronically ill was attributable to the difference in the effects of the variables (explained) and other unobserved factors (coefficients effects or unexplained). The difference in the effects of the variables (explained component) accounted for the remaining 46.2% (–0.012/–0.026) difference in the relative OOP health expenditure gap.

**Table 4:** Blinder-Oaxaca decomposition of the gap in relative OOP health expenditure

	**Coefficient**	**S.E**	**95% CI**	**Coefficient**	**S.E**	**95% CI**
**Differential**						
Not chronic ill	0.050***	0.001	(0.048 to 0.052)			
Chronically ill	0.076***	0.002	(0.073 to 0.080)			
Difference	–0.026***	0.002	(–0.030 to –0.022)			
**Endowments (Explained)**				**Coefficients (Unexplained)**		
**Predisposing factors**						
Gender	–0.000	0.000	(–0.000 to 0.000)	–0.022	0.013	(–0.042 to 0.002)
Age group	–0.003	0.002	(–0.007 to 0.001)	–0.001	0.008	(–0.017 to 0.015)
Marital status	0.001	0.000	(0.000 to 0.002)	–0.046	0.010	(–0.066 to –0.026)
**Enabling factors**						
Education	0.003***	0.001	(0.001 to 0.004)	–0.002	0.004	(–0.010 to 0.006)
Economic status	0.000	0.001	(–0.001 to 0.001)	0.018***	0.005	(0.008 to 0.027)
Employment status	0.001***	0.000	(0.000 to –0.002)	0.018**	0.006	(0.006 to 0.031)
Insurance status	–0.002***	0.000	(–0.002 to –0.001)	0.003**	0.001	(0.001 to 0.005)
**Need factors**						
Self-rated health	–0.005***	0.001	(–0.008 to –0.002)	–0.103***	0.046	(–0.193 to –0.013)
Periodic check-ups	–0.007**	0.003	(–0.009 to –0.004)	0.023***	0.005	(0.012 to 0.033)
_cons				0.098**	0.050	(0.001 to 0.195)
Total	–0.012***	0.002	(–0.017 to 0.007)	–0.014***	0.003	(–0.021 to –0.008)

*95% CI* 95% Confidence Interval

^***^P < 0.01, **P < 0.05

Predisposing factors, including gender, age group, and marital status, were not significant factors that accounted for either the explained or unexplained components. In contrast, the enabling and need factors significantly contributed to the gap in relative OOP health expenditure between the two groups. Under both explained and unexplained components, employment status, insurance status, self-rated health, and periodic check-ups significantly contributed to the gap between the chronically and non-chronically ill. Although education was a significant factor in contributing to the gap under the explained component, it was not an important factor under the unexplained part. Conversely, economic status was not a significant factor that contributed to the gap under the explained component, whereas it was a significant factor under the unexplained component.

## Discussion

This study used data from the national representative family health survey conducted by the General Authority for Statistics in 2018. The study examined the gap and assessed the inequality in relative OOP health expenditure between the chronically and non-chronically ill in the KSA. The study further examined the determinants of such a gap in the context of the KSA. The contribution of the current study lies in the decomposition of the gap in OOP health expenditure between the chronically and the non-chronically ill, which has been missing in the related literature to date. Furthermore, most previous studies limited their analysis of OOP health expenditure to a specific chronic condition or to specific services, making it difficult to generalize the results to a larger population [3, 9, 31]. Applying the Blinder-Oaxaca decomposition analysis, this study provides a clear understanding of the socio-economic inequalities in relative OOP health expenditure and the drivers of such inequalities. This will enable policymakers to develop healthcare financing systems that consider existing inequalities and the influencing factors, in turn minimizing the risk of catastrophic health expenditure among the chronically ill and the poor.

The results showed that, overall, the chronically ill had a higher mean OOP health expenditure of 658.75 SR compared to that of the non-chronically ill with a mean OOP health expenditure of 444.98 SR. The chronically ill spent 7.6% of their income on health compared to 5.0% spent by their non-chronically ill counterparts. This corroborates with previous findings showing that the chronically ill have high OOP health expenditure [32, 33]. Chronic illnesses demand routine medication and sometimes long-term hospitalization, which increase the likelihood of incurring high OOP health expenditure. The high cost of managing chronic illnesses compared with non-chronic illnesses explains the differences in the mean OOP health expenditure reported in this study. Additionally, chronic illnesses universally affect a person’s labour market participation, leading to a reduction in disposable income. This means that the chronically ill spend more on OOP health expenditure as a proportion of their income compared to the non-chronically ill [34].

Moreover, the curves for the chronically and non-chronically ill both showed a pro-poor distribution, meaning that inequalities are concentrated amongst the less well-off. This confirms that the poor spend a higher share of their income on health compared to the well-off, a finding that is dominant in literature [35, 36]. The inequality analysis showed that poor people that also have chronic illness experience even a much higher relative OOP compared to the poor who are not chronically ill. This is not surprising, as poor people within the population have the greatest need for healthcare since they are more likely to suffer from illness and disease [2]. Accordingly, the higher morbidity exhibited among the poor population makes them more likely to be confronted with higher OOP health expenditure. Moreover, since the poorest households have lower spending capacity, any OOP health expenditure constitutes a large proportion of their total expenditure [4]. Nevertheless, pro-poor inequality may have been underestimated given that we applied gross OOP health expenditure which includes payment through private health insurance, unlike the net OOP health expenditure which would have favoured the well-off given that they are more likely to have private health insurance to cover their expenses.

The results of the Blinder-Oaxaca decomposition analysis indicated a statistically significant mean gap of 2.6% in the proportion of OOP health expenditure between the chronically and non-chronically ill. The bulk of this gap is mainly attributable to the effects of the variables (coefficient or unexplained component) alluding to unobserved factors responsible for the difference in the variables. Differences in the various factors drive this gap, including employment status, insurance status, self-rated health status, and periodic check-ups. Households with higher financial capacity are able to purchase health insurance, which reduces OOP health expenditure [20]. However, those with chronic illnesses still face higher OOP health expenditure as they spend more money as a result of frequent use of health services, hospitalization, emergency room visits and grater purchase of routine medication. Similarly, those who perceive their health status to be poor and those who have to do periodic check-ups are likely to face higher OOP payments, especially when compounded by a chronic illness.

Finally, this study established that differences in socio-demographic characteristics, including gender, age, and marital status, did not seem to have any influence on the mean gap under all components, including the explained and unexplained components. This is in contrast to findings in the literature where socio-demographic factors played a key role in influencing OOP health expenditure [7, 11, 37]. Nonetheless, absence of such an influence could indicate that the proportion of people with and without a chronic illness is not significantly different across the socio-demographic characteristics. As such, the influence of these factors remains largely insignificant in determining the gap in OOP health expenditure between the two groups.

This study has several strengths. The data are based on a nationally representative survey and the coverage is not limited to specific types of chronic illnesses or healthcare services. Employing the Blinder-Oaxaca technique, the gap between the chronically and non-chronically ill was decomposed, which has not previously been reported in the related literature in the context of the KSA. Moreover, the main outcome variable of the study was the relative OOP health expenditure, estimated as the percentage of income spent on healthcare, to ensure that the analysis effectively captured proportions instead of levels, which can be greatly misleading. Therefore, the findings are relevant in the design and implementation of a healthcare financing system that accounts for the presence of inequalities between the chronically ill and those without chronic illness.

Nevertheless, there are a few limitations associated with the current study. The estimates of OOP health expenditure could be affected by the structure of the questionnaire, mode of data collection, and recall bias due to the use of self-reported information. The Saudi FHS survey expresses OOP expenditure as the total costs paid for healthcare services and does not discriminate whether any or all of the amount has been reimbursed by private health insurance; hence, caution must be exercised in interpreting the outcome, which represents the general OOP health expenditure rather than the net OOP health expenditure that could also include unobservable reimbursements. Furthermore, the Blinder-Oaxaca decomposition technique has many attractive attributes but does not consider the different distributions of outcomes among the individuals of each group, and the decomposition estimates may vary depending on the choice of reference group. Future studies may have to consider some of these issues in conducting similar analyses.

## Conclusions

Using a recent national representative family health survey conducted by the GaStat, and applying the Blinder-Oaxaca decomposition technique, this study assessed the gap and inequality in the relative OOP health expenditure between the chronically and non-chronically ill in the KSA, and the associated drivers of this inequality. Overall, the results showed that the chronically ill have a high mean relative OOP health expenditure as they spend a larger percentage of their income on health compared to their non-chronically ill counterparts. The study also showed that inequalities are concentrated amongst the less well-off and are higher among those with chronic illness. Therefore, there is a need to further expand strategies such as health insurance, which would act as welfare-equalizing factors when they are equally accessible to the poor and the chronically ill. The results provide evidence for health policymakers to incorporate a social safety net in a health financing system and to prioritize the disadvantaged population to ensure access to health services for all. Furthermore, the government should expand the public healthcare services to meet the high demand for services and to mitigate other barriers to access including long waiting period which pushes people to use private healthcare services. These, together, will mitigate the effect of high OOP healthcare expenditure and prevent impoverishment of vulnerable groups such as the poor and those with chronic illness.

## Data Availability

The datasets generated and/or analysed during the current study are not publicly available due to privacy, confidentiality, and other restrictions. Access to data can be gained through the General Authority for Statistics in Saudi Arabia via https://www.stats.gov.sa/en.
